# Everolimus in the Treatment of Neuroendocrine Tumors: Lights and Shadows

**DOI:** 10.3390/biomedicines13020455

**Published:** 2025-02-12

**Authors:** Bianca Medici, Eugenia Caffari, Yuri Maculan, Stefania Benatti, Federico Piacentini, Massimo Dominici, Fabio Gelsomino

**Affiliations:** Department of Oncology and Hematology, Division of Oncology, University Hospital of Modena, 41124 Modena, Italy; bia.medici31@gmail.com (B.M.); eugeniacaffari@gmail.com (E.C.); yuri.maculan94@gmail.com (Y.M.); stefania.benatti@unimore.it (S.B.); federico.piacentini@unimore.it (F.P.); massimo.dominici@unimore.it (M.D.)

**Keywords:** everolimus, neuroendocrine tumors, controversies, sequencing, combination, markers

## Abstract

Neuroendocrine tumors (NETs) comprise a heterogeneous group of neoplasms that originate from neuroendocrine cells, characterized by their ability to secrete hormones and peptides. Once considered rare, the incidence of NETs has steadily increased due to improved diagnostic modalities. The therapeutic landscape is multifaceted, ranging from surgery for localized disease to pharmacological interventions for advanced cases. However, the absence of robust predictive biomarkers precludes patient stratification and optimization of therapy. Everolimus, an oral mTOR inhibitor, has emerged as a key therapeutic agent due to its dual role in inhibiting cell proliferation and angiogenesis. Nevertheless, challenges such as resistance mechanisms, toxicity and optimal treatment sequencing remain unresolved. This article provides a comprehensive review of the role of everolimus in the management of NETs, focusing in particular on unresolved issues, from the absence of predictive biomarkers to the unavailability of defined guidelines for determining the correct therapeutic sequence.

## 1. Introduction

Neuroendocrine tumors (NETs) represent a large and heterogeneous group of neoplasms originating from neuroendocrine cells throughout the organism. Although in the past they were considered relatively rare, their incidence and prevalence are gradually increasing globally, particularly in USA, Canada and Norway [1].

They can secrete an excess of hormonal substances, which are responsible for some peculiar clinical syndromes, the most frequent of which is represented by carcinoid syndrome.

In the localized stage, surgery is the only curative therapy and should be considered the treatment of choice, although in peculiar cases, watch and wait can be deemed as an acceptable strategy. However, the majority of NETs are diagnosed at an advanced stage in which there are currently many therapeutic options available, although their specific use and sequencing remain controversial [2].

One of the pivotal drugs in the treatment of these malignancies for more than a decade has been everolimus, an inhibitor of the mammalian target of rapamycin (mTOR), which blocks the pathway through the bond it forms with the protein known as FKBP-12, leading to the formation of a complex that blocks the activity of mTORC1, one of the two multiprotein complexes that constitute and enable mTOR function [3].

Although this drug became part of the therapeutic armamentarium in NETs for many years, several unsolved questions remain.

In this manuscript, we will do a timely and up-to-date review of the literature about the use of everolimus in the setting of NETs, its mechanisms of action, its pre-clinical and clinical development, the potential predictive biomarkers, which have yet to be explored, and its ideal positioning in the therapeutic algorithm of NETs.

## 2. mTOR Pathway

**Figure 1:**
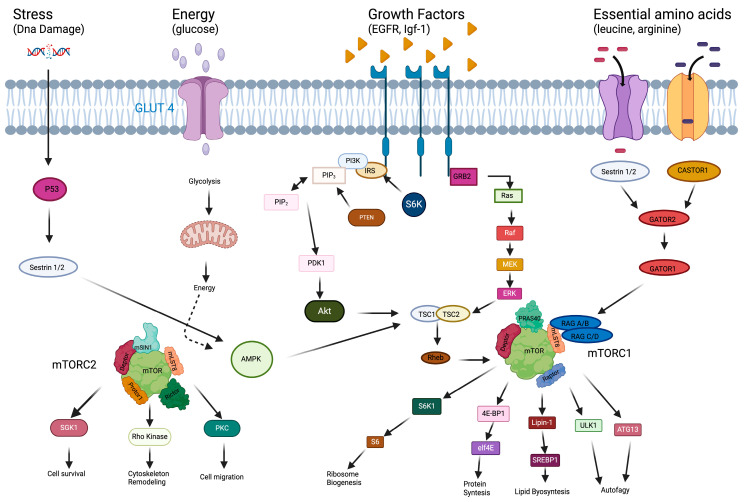
The mTOR pathway with extracellular and intracellular signals.

The mammalian target of the rapamycin (mTOR) pathway integrates both intracellular and extracellular signals and acts as a central regulator of cellular proliferation, growth, survival and metabolism (Figure 1).

The mTOR protein is a serine–threonine kinase and a member of the phosphoinositide 3-kinase (PI3K)-related kinase family. It serves as the catalytic subunit in two distinct protein complexes: mTORC1 and mTORC2 [4].

Overall, mTORC1 consists of five proteins: mTOR, raptor, GβL, deptor, and PRAS-40. It plays a vital role in regulating metabolism, growth and cell proliferation by activating various anabolic processes, such as protein and lipid synthesis, while suppressing catabolic activities like autophagy [5].

mTORC1 specifically enhances protein synthesis through the phosphorylation of two key targets: the eukaryotic initiation factor 4E (eIF4E)-binding protein 1 (4E-BP1) and the p70 ribosomal S6 kinase 1 (S6K1). The phosphorylation of 4E-BP1 prevents it from binding to eIF4E, allowing eIF4E to drive cap-dependent translation. In addition, the activation of S6K1 boosts mRNA synthesis, protein translation and ribosomal RNA transcription, supporting the production of new ribosomes [6]. mTORC1 also promotes lipid production by activating the transcription factors SREBP1 and PPARγ, which regulate genes involved in lipid metabolism [4].

In contrast, it suppresses autophagy, a catabolic process responsible for breaking down cellular components and recycling proteins. mTORC1 achieves this by phosphorylating ULK1 and ATG13, two key proteins essential for initiating autophagy [7].

mTORC1 responds to four primary extracellular signals: growth factors, amino acids, oxygen levels and energy status, integrating these inputs to regulate cellular functions.

Growth factors like insulin, along with high levels of oxygen, amino acids or ATP, trigger the phosphorylation of TSC1 and TSC2, which together form the tuberous sclerosis complex (TSC). This complex phosphorylates Rheb, a protein that directly activates mTORC1. The increased activity of mTORC1 then promotes cell growth [8,9].

Moreover, mTORC2 consists of six proteins: mTOR, Rictor, mSIN1, Protor-1, GβL and deptor. Though less well understood than mTORC1, mTORC2 plays a key role in cell proliferation, survival and cytoskeletal organization by directly activating the PI3K/AKT pathway [10]. AKT is a crucial protein for promoting cell proliferation and survival as it phosphorylates multiple downstream effectors that regulate these processes. To become active, AKT must be phosphorylated at two specific sites: Ser308, phosphorylated by PDK1, and Ser473, phosphorylated by mTORC2 [11].

In conclusion, the mTOR pathway activates multiple signal transduction pathways, including PI3K/AKT, TSC1/TSC2/Rheb, LKB1/AMPK and VAM6/Rag GTPases. mTOR plays an important role in regulating cell proliferation, growth, apoptosis and autophagy by processing and integrating complex intra- and extracellular signals.

Given its central role, it is evident how the dysregulation of the mTOR pathway can be a key factor in tumorigenesis.

## 3. Rationale and Mechanism of Action of Everolimus in NETs

Like Rapamicin, everolimus binds FKB-12, creating a complex that binds mTOR within the mTORC1 complex and blocking downstream signaling with inhibitory action. mTORC1 represents a very important target for antiproliferative therapies because it is involved in the processes of cell survival and proliferation, controlling the processes of mRNA transcription and cell cycle progression. The inhibition of mTORC1 induces the downregulation of the downstream effector 4E-BP and S6K1. 4E-BP is involved in the initial stages of the mRNA translation process by recruiting cell cycle regulators (cyclin D1 and ornithine decarboxylase) and transcription factors (c-MYC and HIF-1α). S6K1 usually negatively regulates IGF-1 receptor signaling through the phosphorylation of IRS-1, which leads to its proteasomal degradation. However, the stabilization of IRS-1 induces the hyperphosphorylation of the AKT downstream of mTORC2 and, consequently, the activation of VEGF with the induction of tumor angiogenesis. This may explain why many tumors are intrinsically resistant to mTOR inhibitors [12,13].

The mTOR pathway plays a crucial role in the pathogenesis of NETs. These neoplasms are usually sporadic, but they can also arise within hereditary genetic syndromes [14]. Increased mTOR activity was detected in both settings.

In the context of inherited genetic syndromes, neurofibromatosis type 1 and tuberous sclerosis show autosomal dominant mutations that inactivate suppressor genes NF1, TSC1 and TSC2 [14]. The loss of function of these genes leads to a constant activation of the mTOR pathway and its proliferative signals, predisposing to the development of midgut and pancreatic NETs (pNETs) [15].

In neurofibromatosis type 1 and tuberous sclerosis, there is an altered expression of the TSC complex, which would suggest a higher incidence of neuroendocrine neoplasms (NENs) as a few examples are documented in the literature. However, the actual incidence is modest, rendering this contradiction unclear [16,17].

Moreover, multiple endocrine neoplasms (MENs) are a group of autosomal dominant diseases characterized by a higher prevalence of neuroendocrine tumors. The most prevalent forms are MEN-1 and MEN-4, with the incidence of neuroendocrine neoplasms (NEN) varying from 30% to 90% among affected people [18]. These patients were included in the RADIANT studies; however, due to their limited number, no subgroup analysis was conducted. The literature lacks randomized trials assessing the efficacy of everolimus in this patient subgroup; nonetheless, a limited number of case reports indicate some effectiveness of the medicine [19].

Among the sporadic cases, Y. Jiao et al. conducted a whole exome sequencing of 68 non-familial pNETs, and they detected the presence of mutations affecting the mTOR pathway components TSC2, PTEN and PI3K in 14% of the tumors, with a reduction in the suppressive action of these genes [20].

Focused on pNETs, Missiaglia et al. discovered that the downregulation of TSC2 and PTEN affects many of the sporadic cases with percentages of 35% and 60% of patients, respectively, and low levels of these components may help tumor cells to escape the mTOR inhibition [21].

Regarding prognostic significance, Qian et al. found that mTOR activation is usually correlated with a higher proliferative index [22]. Consequentially, the upregulation of mTOR and its downstream components seem to be associated with a more aggressive tumor behavior, with a higher tendency for distant metastases and a shorter overall survival [21].

Based on these data, many in vitro and in vivo studies have been conducted to demonstrate the efficacy of mTOR inhibitors in the treatment of metastatic NETs to overcome resistance to apoptosis and uncontrolled cell expansion caused by the hyperactivation of the mTOR pathway [21,23,24].

Everolimus was tested for its antiproliferative action on a human pNET cell line by Zitzmann and colleagues. This drug substantially inhibited cell growth, arresting the cell cycle in G0/G1 and inducing apoptosis [23].

Kasajima et al. found that mTOR inhibitors depend on target activation and downstream effector phosphorylation [25].

Furthermore, an Italian study isolated bronchial carcinoid cell lines that exhibited low levels of mTOR, AKT and ERK1/2 and suggested that these phenotypes conferred resistance to mTOR inhibitors [26].

It is also interesting to note that higher levels of mTOR expression were found in foregut tumors compared to midgut tumors [25].

From these considerations, we can hypothesize that mTOR pathway components, which are differentially expressed according to the primary site, may potentially represent biomarkers of response to treatment with mTOR inhibitors, although literature data about this topic are controversial and need to be further validated.

## 4. Clinical Development of Everolimus in NETs: RADIANT Studies

Overall, several phase II and III trials have been conducted to study the efficacy of everolimus in inoperable or metastatic NETs; here, worth mentioning are the RADIANT studies (Table 1).

RADIANT-1 is a phase II study involving 160 patients with low to intermediate-grade metastatic pNETs, aiming at evaluating the efficacy of everolimus in patients progressive to prior chemotherapy. Patients were divided into two cohorts and received everolimus 10 mg daily with or without octreotide, an octapeptide analogue of somatostatin that mimics the pharmacological effect of somatostatin. The primary endpoint was objective response rate (ORR) in the first group (everolimus), while secondary endpoints included ORR in the second group (everolimus and octreotide) and progression-free survival (PFS), overall survival (OS) and the safety and duration of response in both study arms. In the group treated with octreotide extended-release, PFS was 16.7 months, while OS was not reached at the time of data cutoff with a RR of 4.4%. In the group treated with everolimus monotherapy, PFS was 9.7 months, and OS was 24.9 months with a ORR of 9.6% [27].

Based on RADIANT-1 data, RADIANT-2 and RADIANT-3 trials were launched simultaneously.

The RADIANT-2 was a phase III double-blinded randomized trial, which studied the efficacy of everolimus in combination with octreotide LAR in 429 patients with advanced low to intermediate-grade functional NETs with gastrointestinal, pulmonary or unknown origin. Patients were randomized 1:1 to everolimus 10 mg daily versus placebo, both in combination with octreotide LAR 30 mg every 28 days. The primary endpoint was PFS that was 11.3 months in the placebo group, versus 16.4 months in everolimus group. From a clinical point of view, this result was quite relevant. However, it did not translate into a statistically significant difference [28].

The RADIANT-3 was a multicenter, double-blind, randomized phase III study of 410 patients with low to intermediate-grade advanced pNET who were randomized 1:1 to everolimus 10 mg daily vs. placebo. The primary endpoint was PFS, which was considerably longer in the everolimus arm (11 months versus 4.6 months). Considering that the study design allowed crossover in the case of documented radiological progression according to the RECIST criteria, OS did not differ between the two groups [29,30].

Finally, the RADIANT-4 was a phase III, double-blind, randomized study that involved 302 patients with advanced, progressive, well-differentiated, nonfunctional lung or gastrointestinal neuroendocrine tumors. Previous treatments with somatostatin analogues were accepted but not continued in the study. Patients were randomized on a 2:1 to everolimus 10 mg daily versus placebo. The primary endpoint was PFS that was 11 months in the everolimus arm versus 3.9 months in the placebo group [31].

Based on these data, on December 2012, the European Medicines Agency approved everolimus for adult patients with progressive, well or moderately differentiated, inoperable or metastatic neuroendocrine tumors of pancreatic origin.

Thereafter, on February 2016, the U.S. Food and Drug Administration approved everolimus for the treatment of adult patients with progressive, well-differentiated non-functional, neuroendocrine tumors of gastrointestinal or lung origin with unresectable, locally advanced or metastatic disease. 

## 5. Toxicity Profile

Everolimus presents a distinct toxicity profile that differs from traditional chemotherapy. Overall, drug-related side effects occur in 40–50% of patients treated with everolimus, including stomatitis, rash, fatigue, diarrhea, pneumonitis and metabolic abnormalities, such as hyperglycemia and dyslipidemia. Globally, everolimus should be avoided in patients with uncontrolled diabetes, pulmonary disease or systemic infections and used cautiously in those with uncontrolled dyslipidemia [32].

Treatment with everolimus requires strict clinical and laboratory surveillance to promptly manage potentially serious side effects and ensure patient safety, in particular, renal function, hematological parameters (severe toxicity is often a hematological side effect, for example, thrombocytopenia) and glucose/lipids must be monitored during the treatment [33].

In RADIANT-1, common side effects included stomatitis (62%), rash (37%) and fatigue (31%). Grade 3–4 toxicities were rare, with pulmonary events like pneumonitis observed in 8–12% of cases. In RADIANT-2, the most frequent side effects reported in patients receiving everolimus plus octreotide LAR were rash (53%), stomatitis (58%), diarrhea (26%) and dysgeusia (26%). Finally, in the RADIANT-3 trial, stomatitis (64%), rash (49%), diarrhea (34%) and infections (23%) were more frequent in the everolimus group compared to placebo. Pneumonitis was seen in 1% of patients. Rare events included pulmonary tuberculosis, bronchopulmonary aspergillosis and hepatitis B reactivation [34].

Similarly, in a systematic review and meta-analysis of patients treated with everolimus for different indications, the most common adverse effects were stomatitis (43.2%), characterized by painful oral ulcers. Grade 3 or 4 adverse events were less common (6.8%), with fatigue (23.7%), diarrhea (22.3%), anemia (24.4%) and thrombocytopenia (21.8%) being the most common toxicities. As regards metabolic effects, hyperglycemia occurred in 16.9% of patients and hypercholesterolemia in 20.2%. Overall, most adverse events were grades 1 and 2, easier to manage as compared to chemotherapy-related events, which more frequently reached grades 3 or 4 [35].

As previously illustrated by literature data, stomatitis and ulcerations are common side effects, and they are often a dose-limiting side effect. Topical treatments are preferred, avoiding irritating mouthwashes or antifungal agents, unless a fungal infection is documented [32].

Among noticeable side effects is non-infectious pneumonia, which is a potential complication with a reported incidence of 12% among patients. This condition can range from asymptomatic to severe and, in rare cases, may be fatal. It is essential to consider this diagnosis in patients presenting with respiratory symptoms such as dyspnea, cough or hypoxia after excluding other infectious or neoplastic causes [34]. The management of non-infectious pneumoniae depends on severity: in mild cases, treatment may continue; in moderate or severe cases, temporary or permanent discontinuation may be necessary, with the possible use of corticosteroids [36].

Moreover, as an immunosuppressant, everolimus increases the risk of bacterial, fungal and viral infections, including severe forms such as aspergillosis or viral reactivations. Indeed, it is crucial to identify and treat pre-existing infections before initiating therapy and to closely monitor signs of infection during treatment. Invasive fungal infections require a permanent discontinuation of the drug.

Furthermore, everolimus may cause renal failure, including fatal cases. Regular monitoring of renal function is recommended, particularly in patients with pre-existing risk factors.

As concerns dyslipidemia (with increases in cholesterol, LDL and triglycerides and a possible elevation in HDL), one of the most peculiar side effects of the drug, the cause of this effect is to be found in the fact that the mTOR pathway regulates cellular growth and metabolism and inhibitors like everolimus disrupt anabolic processes, reduce lipogenesis and impair glucose homeostasis. Hyperglycemia occurs in 10–50% of patients, and it is dose-dependent and more pronounced in cancer therapy than in immunosuppressive doses [37].

However, in some cases, everolimus-induced hyperglycemia can be useful to control blood glucose levels. M. Asayama et al. highlighted that hyperglycemia caused by everolimus may have a beneficial effect in the treatment of refractory and life-threatening hypoglycemia in patients with unresectable insulinoma [38]. The French group coordinated by V. Bernard selected 12 patients with insulinoma treated with everolimus, obtaining a rapid normalization of blood glucose values in 11 patients. It is important to underline that this result was obtained without a morphological reduction of the tumor mass [39]. Therefore, the cause is not to be found in the pathological response to everolimus but in other associated mechanisms that act simultaneously. Firstly, everolimus promotes peripheral insulin resistance in adipocytes and skeletal muscle by inhibiting AKT phosphorylation. Secondly, it reduces insulin secretion by pancreatic β-cell and increases hepatic gluconeogenesis and blood glucose release as a result of mTORC2 destruction [40].

These metabolic disruptions necessitate a multidisciplinary approach, involving oncologists and endocrinologists, to optimize therapeutic benefits while mitigating risks. The intricate interplay between the mTOR pathway and metabolic processes exemplifies the challenge of balancing efficacy against systemic impacts, particularly in vulnerable cancer patients.

Side effects and effectiveness are also influenced by any medications the patient takes outside the treatment for NET.

The interindividual pharmacokinetic variability of everolimus can be explained by the different activities of the drug efflux pump P-glycoprotein and metabolism by cytochrome P450 (CYP) 3A4, 3A5 and 2C8. The critical role of the CYP3A4 system in the biotransformation of everolimus leads to drug–drug interactions with other drugs metabolized by this cytochrome system [41].

In patients with hepatic impairment, the apparent clearance of everolimus is significantly lower than in healthy volunteers; therefore, the dosage of everolimus should be reduced by half in these patients [42].

Everolimus interferes with several drugs. For example, the dose of everolimus in combination with tacrolimus must be higher than in combination with cyclosporine to achieve a given blood level of everolimus [43].

The concomitant administration of strong CYP3A4 inhibitors (e.g., ketoconazole, itraconazole, ritonavir, erythromycin and fluconazole) or P-gp inhibitors should be avoided, as should the concomitant administration of moderate CYP3A4 or P-gp inhibitors [42].

The pharmacokinetics and pharmacodynamics of everolimus have been analyzed by a number of studies, particularly in metastatic breast cancer. One study, conducted in 22 metastatic breast cancer patients treated with everolimus and exemestane, showed that everolimus only partially inhibited mTOR activity. The study suggests that pharmacokinetically guided the dosing of everolimus, targeting a trough concentration below 17.3 ng/mL, may improve safety, and potentially efficacy, and warrants further investigation [44]. Another study of 18 Japanese breast cancer patients taking everolimus found significant inter-individual variation in everolimus pharmacokinetics. In particular, higher trough concentrations were associated with dose-limiting toxicities. Patients with trough concentrations between 10 and 20 ng/mL experienced significantly longer progression-free survival [45].

In conclusion, everolimus offers a safer alternative to traditional chemotherapy, with most adverse effects being manageable and mild. However, its unique safety profile necessitates tailored monitoring and intervention strategies to maximize therapeutic benefits while minimizing risks.

## 6. Controversies

### 6.1. Predictive Biomarkers

Predictive biomarkers present an essential advantage over prognostic biomarkers in the era of personalized medicine that is only starting. Identifying patients likely to respond to particular therapies helps to create customized treatment programs that guarantee the effective use of resources, lower negative effects and improve outcomes. As of now, predicting everolimus efficacy in NETs is not supported by any validated biomarkers.

Using tissue samples from 24 patients treated with everolimus, our group investigated the expression levels of phosphorylated mTOR (p-mTOR) and p70S6-kinase (p-S6K), both markers of motor pathway activation. Patients that expressed these markers positively showed notably higher PFS and OS. Patients positive for both p-mTOR and p-S6K had a median PFS of 18.2 months and an OS of 39.9 months; those negative for these markers had a median PFS of 13 months and an OS of 32.4 months. This statistical relevance emphasizes the potential of mTOR pathway activation as a prognostic marker [46].

These results imply that evaluating mTOR pathway activity could help doctors to pinpoint patients more likely to benefit from everolimus treatment. Stratifying patients depending on biomarker expression helps doctors to more precisely customize treatment plans, so perhaps enhancing patient outcomes.

In a cohort of 195 NET patients, another study examined the expression of several mTOR pathway components, including mTOR itself and its downstream targets (such p-RPS6KB1 and p-RPS6). High expression levels of these components linked to negative clinical outcomes, implying their possible use as prognostic biomarkers. Particularly, highly phosphorylated RPS6KB1 (p-RPS6) and RPS6 (p-RPS6) were linked to noticeably lower overall survival rates, suggesting that mTOR pathway activation might be involved in tumor aggressiveness. Patients showing high levels of these markers may gain from focused treatments meant to block the mTOR pathway [22].

Furthermore, gathered in a retrospective analysis of 53 patients with metastatic NETs who received everolimus were a range of clinical, biological and histological data including information on cholesterol levels and the expression of p-p70S6K, a fundamental protein engaged in the mTOR pathway. Patients with hypercholesterolemia brought on during everolimus treatment had much longer PFS. This implies that hypercholesterolemia might be a first sign of a positive drug reaction. Conversely, shorter PFS linked to an overexpression of p-p70S6K in tumor cells suggested that it might be a marker of resistance to everolimus [47].

These results point generally to a possible function of mTOR pathway expression in predicting everolimus efficacy. Nonetheless, given the retroactive character of the studies and the small sample size, the heterogeneity of the population enrolled demands more validation.

Apart from molecular markers, radiological parameters are also under investigation to find elements influencing response to everolimus.

With an eye toward perfusion computed tomography (P-CT), a useful tool for forecasting early response to anti-angiogenic treatments in pancreatic neuroendocrine tumors, D’Onofrio et al.’s P-CT specifically measures tissue vascularization and perfusion, hence it is a possible biomarketer for treatment response; changes in perfusion parameters, such as blood flow and blood volume, can indeed correlate with response to anti-angiogenic therapies including bevacizumab and everolimus. By inhibiting endothelial and stromal cell growth and lowering VEGF release, everolimus has indirect anti-angiogenic effects that can change tumor vascularization including higher vascular permeability and blood leakage. Better response to everolimus treatment was linked in this study to a rise in blood volume and a drop in peak enhancement intensity on P-CT. The rather small sample size of the study limited the strength of the conclusions by virtue of this. Still, more research is required to verify P-CT’s predictive worth in a bigger patient population [48].

In order to investigate the effectiveness of a radiomic approach in predicting patient outcomes (PFS and OS) for 25 patients with metastatic NETs, mostly pancreatic and ileal, a retroactive study was performed. Before starting everolimus treatment, contrast-enhanced CT scans were obtained; statistical tests were then used to compare the radiomic features of responders and non-responders. Ten patients were non-responsive (median PFS: 4.5 months; median OS: 23 months), while fifteen patients were responders (median PFS: 25 months; median OS: 29 months). In both internal and external validation cohorts, two radiomic characteristics—correlation and Imc1—showered notable variations between the two groups (*p* < 0.05) and showed strong predictive performance (AUC: 0.84–0.90; *p* < 0.0001).

In conclusion, the radiomic analysis of pre-treatment CT images seems to be a non-invasive biomarker for predicting PFS and OS in patients with metastatic NETs treated with everolimus. However, further prospective validation studies are warranted to establish the clinical utility of this approach [49].

### 6.2. Treatment Sequencing and Combination

Everolimus is an effective therapy in second or further lines for patients with GEP-NETs and lung NETs.

Several therapeutic options are now available for NET patients. Randomized phase III trials evaluating somatostatin analogs (SSAs), targeted drugs like everolimus and sunitinib and peptide receptor radionuclide therapy (PRRT) have influenced the current treatment recommendations. On the other hand, prospective randomized trials on surgical, ablative and chemotherapy treatments have been limited.

Due to a lack of head-to-head comparison studies, optimal treatment sequencing has not been established. In cases of multifocal tumor progression, there is limited evidence on which treatment options should be prioritized. In clinical practice, treatment should be individualized based on factors like tumor type and primary site, grade, somatostatin receptor (SSTR) expression, disease extent and patient characteristics such as age, performance status, comorbidities, symptoms and preferences [50,51,52].

Instead, studies focusing on the sequence of various treatments and particularly on when to use everolimus are varied and not always in agreement. This, therefore, underscores the importance of discussing these clinical cases in a multidisciplinary setting.

Recent guidelines generally recommend everolimus as a second-line therapy for NETs, following somatostatin analogs, which represent the first-line treatment, at least for the majority of NETs [36].

Other treatments, such as sunitinib (for pancreatic NETs), PRRT, chemotherapy and liver-directed therapies, are also viable options [36].

The association with IGF-1 inhibitors could represent a further possible alternative to enhance the effect of mTOR inhibitors. Through the IGF-1 receptor, IGF-1 leads to the activation of the PI3K/Akt pathway, similarly to mTOR, sending anti-apoptotic and proliferative signals to the cell. Therefore, it has been suggested that the combination of mTOR inhibitors and IGF-1 inhibitors may achieve a synergistic effect to overcome secondary resistance to everolimus [53]. A phase I study combining everolimus and octreotide with cixutumumab (anti-IGF-1R antibody) has been conducted in the treatment of well-differentiated NETs, with a significant toxicity profile [54]. Other phase I and II trials combining IGF-1R and mTOR inhibitors have found more promising results in the treatment of sarcomas, breast cancer and acute myeloid leukemia. However, further clinical studies are needed [55,56].

#### 6.2.1. Sequencing

Treatment options for metastatic GEP-NETs are limited. Standard therapies include somatostatin analogs and targeted drugs like everolimus and sunitinib, which generally stabilize disease rather than induce remission, chemotherapy and PRRT.

The COMPETE study is a prospective, randomized, controlled, open-label, multi-center phase III trial evaluating 177Lu-Edotreotide PRRT versus everolimus in patients with inoperable, progressive, somatostatin receptor-positive GEP-NETs [57].

Conducted across 40 centers in 11 countries, it aims to recruit 300 patients. Participants will be randomized to receive either up to four cycles of 177Lu-Edotreotide RLT (7.5 GBq per cycle) every 3 months or 10 mg of everolimus daily for 24 months. The primary endpoint is PFS, with secondary endpoints including ORR, median duration of disease control, safety, tolerability, dosimetry measures, OS and quality of life. Disease progression and hepatic tumor burden will be assessed using MRI and/or CT based on RECIST 1.1 criteria [57].

Solid data on a direct comparison between PRRT and targeted agents as second-line therapy in patients with progressive disease after SSA are currently not available.

Pusceddu et al. compared the effectiveness of upfront PRRT versus chemotherapy or targeted therapy in patients with advanced GEP-NETs who had progressed after somatostatin analogue treatment. Patients who received upfront PRRT had significantly longer PFS compared to those who received chemotherapy or targeted therapy. This benefit was consistent across different tumor characteristics, except for high-grade tumors and those with a high proliferation rate. On the other hand, there was no significant difference in OS between the two treatment groups [58].

A multicenter, retrospective study evaluated treatment sequences in advanced, well-differentiated pNETs based on PFS and OS. Among 201 patients, most had grade 2 tumors, with 48.8% having undergone primary tumor resection. In the first-line setting, patients received standard-dose SSA alone, SSA and RLT, temozolomide or SSA and targeted therapy (everolimus or sunitinib). Patients with G1 tumors, those who had primary surgery, and those treated with PRRT had significantly longer PFS. Multivariate analysis confirmed PRRT was independently associated with improved outcomes. In second-line treatments, PRRT again showed better PFS compared to other options. OS was longest in patients who underwent primary surgery, had a Ki67 index ˂ 10%, without extrahepatic disease, and followed an SSA-PRRT sequence. Overall, PRRT and the SSA-PRRT sequence were linked to the best survival outcomes, particularly in patients with Ki67 < 10% [52].

Another study, the SEQTOR study aimed to determine the optimal treatment sequence between everolimus and chemotherapy (streptozotocin-5FU) in patients with pNETs. Due to slow patient enrollment, the study could not compare the full treatment sequences and shifted its primary endpoint to PFS at 12 months for first-line treatment (12-m PFS1). Both arms had similar 12 m PFS1, but the chemotherapy group showed a significantly higher ORR compared to everolimus (30% vs. 11%). Based on these findings, STZ/5-FU should be considered a valid option as a first-line treatment for patients with pNETs and good performance status, in particular, when tumor shrinkage is required. Primary tumor resection was also associated with longer survival, though the role of surgery in metastatic neuroendocrine tumors remains controversial due to the heterogeneity of these tumors and limited clinical evidence [59].

Earlier retrospective studies suggested that nonconventional-dose SSAs might offer better outcomes as a second-line treatment compared to later-line treatments. However, the phase II CLARINET FORTE study, which evaluated doubled-dose lanreotide (120 mg every 2 weeks) in 99 patients with advanced G1 or G2 midgut or pNETs after progression on the standard dose, did not clearly confirm these findings. The study reported the median PFS of 8.3 months for midgut NENs and 5.6 months for pancreatic NETs, with a 45% disease control rate, mostly through disease stabilization. A systematic review and meta-analysis also found similar disease control rates with increased SSA doses. In contrast, targeted agents like everolimus and sunitinib have shown consistent efficacy in progressive GEP-NENs, providing an 11–16-month PFS advantage over placebo based on earlier phase III trials [32,60].

#### 6.2.2. Combination

MTOR inhibitors typically stabilize disease rather than induce tumor regression. To achieve tumor regression, there have been efforts to combine mTOR-targeted therapies with other drugs to enhance cytotoxic effects. While rapalogs inhibit mTOR, they can activate upstream signaling pathways like Akt, which may limit their antitumor efficacy. Preclinical models have not consistently supported this synergy, and clinical trials have yielded mixed results [51].

Both VEGFR and mTOR inhibitors are effective in treating neuroendocrine tumors and combining these agents could enhance their effectiveness.

Therefore, the combination of everolimus with bevacizumab has been studied in a phase II study by Yao et al. The CALGB 80,701 trial compared everolimus alone with the combination of everolimus and bevacizumab in 150 patients with advanced pNETs. The combination therapy resulted in significantly higher response rates (31% vs. 12%) and a slight increase in PFS (16.7 vs. 14 months), although this difference was not statistically significant (HR: 0.80; *p* = 0.12) [61].

A phase I trial by Chan et al. found that the combination of everolimus and sorafenib showed activity, with 62% of patients experiencing tumor shrinkage, but concerns about toxicity may limit its use [51].

Research in animal models has demonstrated synergism in the combined use of everolimus and PRRT [62,63]. M. Grzmil et al. highlighted that everolimus-induced mTORC1 inhibition promotes the internalization of the radioligand [177Lu]Lu-PP-F11N by upregulating the target receptor on the tumor cell and acting as a radiosensitizer in NENs. Thus, preclinical models suggest a potential for the improvement of PRRT and nuclear imaging, when combined with mTOR inhibitors [63]. Based on these premises, A. Aljubran and colleagues enrolled 11 patients affected by G1–G2 NETs of all origins and subjected them to a combined treatment of everolimus 10 mg daily and 177Lu-DOTATATE at an interval of 8 weeks. However, the trial was stopped early due to disease progression or excessive toxicity in all patients. Currently, this treatment combination does not appear to be possible, but studies with a larger number of patients and lower doses of everolimus are needed [64].

A phase 2 study evaluated the efficacy and safety of combining everolimus and temozolomide as a first-line treatment for metastatic high-grade gastroenteropancreatic neuroendocrine neoplasms with a Ki-67 index ≤ 55%. Patients received everolimus daily and temozolomide biweekly. The study found a 65% disease control rate at 6 months, with a 30% response rate. Median PFS was 10.2 months and median OS was 26.4 months. The treatment was more effective in NET G3 patients compared to NEC. Toxicity was significant, with 43% experiencing grade 3 and 38% grade 4 adverse effects, but the quality of life remained stable [65].

In a study comparing the effects of combining pasireotide, a second-generation somatostatin analog, with everolimus versus everolimus alone for patients with advanced pNET, the combination therapy did not show a significant benefit over everolimus alone in terms of PFS. While the combination therapy showed a higher response rate (20.3% versus 6.2%), the overall disease control rate and median OS were comparable between the two groups. The combination therapy was associated with a higher incidence of grade 3 or 4 fasting hyperglycemia. The study suggests that adding pasireotide to everolimus does not improve PFS, and further research is needed to understand how somatostatin analogs affect tumor growth in NETs [65].

Moreover, there are numerous ongoing studies exploring various agents alone or in combination with everolimus for treating NETs. Given the high vascularity of NETs, some of these agents target vascular pathways. For instance, fosbretabulin is a vascular disrupting agent that targets blood vessels, while X-82 and vatalanib are both oral drugs targeting VEGFRs and platelet-derived growth factor receptors. Additionally, trials are investigating agents that target components of the mTOR pathway or related pathways to address tumor escape mechanisms. These include the following [51]:

- Cixutumumab and R1507: monoclonal antibodies targeting IGF receptor 1.
- Erlotinib: an EGFR inhibitor that may disrupt feedback loops stimulating upstream signaling like PI3K and Akt.
- Alpelisib: an oral inhibitor of the PI3K catalytic subunit p110α.
- BEZ235: a dual PI3K/mTOR inhibitor.
- Sapanisertib: a drug blocking both mTORC1 and mTORC2.

Considering that everolimus is involved in metabolic processes, particularly glucose and lipid processes, some clinical trials have also investigated the association between treatments for NET and metformin.

In particular, Kurita et al. found that the use of everolimus combined with metformin was associated with an increase in PFS in their study on the efficacy of everolimus in pNEN (HR 0.29; 95% CI: 0.09–0.97; *p* = 0.044). However, no statistically significant advantage was found in the association with insulin. (HR 0.92; 95% CI: 0.47–1.78; *p* = 0.799). The mechanism of action of the drug involves that metformin reduces blood sugar, insulin and IGF-1 levels. In particular, by inhibiting mTOR and the IGF-1 oncogene activation pathway, metformin may have a synergic effect with everolimus. This combination can, therefore, be considered beneficial in diabetic patients when compared to insulin use [66].

In addition, Pusceddu, in the phase Ib trial MetNET2, investigated the safety and efficacy of metformin in combination with lanreotide in patients with advanced well-differentiated neuroendocrine tumors. The study demonstrates the safety and potential antitumor activity of metformin, regardless of diabetes status [67].

## 7. Everolimus Beyond the Approved Indications

The fifth edition of the WHO classification of tumors recognizes NET G3 and NEC as separate entities [68]. Both have a Ki67 index > 20% and poorer prognoses as compared to low and intermediate-grade NETs. However, NECs are recognized because they usually have a Ki67 > 55% and extensive necrotic areas. In addition, NECs tend to have a faster and more aggressive disease course [69].

While the therapeutic algorithm of NET G3 is similar to that chosen for the lowergrade forms, the first-line treatment for NECs is platinum-based chemotherapy, and the second-line approaches are more discouraging, with poor ORR and PFS despite various combination chemotherapies [70,71].

Regarding everolimus, many studies have demonstrated its efficacy in the treatment of well-differentiated G1 and G2 NETs [30,72,73]. However, its role in the management of well-differentiated G3 NETs and poorly differentiated NECs is debated, and data are very scarce. In fact, RADIANT trials were conducted before the formal recognition of NET G3 as a separate entity in the WHO classification (2017 for pancreatic NENs and 2019 for GEP NENs).

Thanks to the RADIANT trials, everolimus has been introduced into the clinical practice of NETs. Nevertheless, the inclusion criteria of these trials allowed for the evaluation of the efficacy of the drug only in well-differentiated advanced neuroendocrine tumors grade 1 or 2, excluding more aggressive tumors [73].

A French study evaluated the anticancer activity of everolimus in preclinical models of high-grade GEP NENs based on the concept that high levels of phosphorylated mTOR are frequently detected in NECs. It has been observed that everolimus strongly inhibited tumor development both in vitro and in vivo, suggesting its possible therapeutic use in GEP NEC [74].

Afterwards, in clinical settings, few studies regarding NECs have been published and with contradictory results.

Despite promising outcomes described in individual cases, larger retrospective and experimental studies have provided disappointing results in the use of everolimus in G3 NENs.

A retrospective study conducted on pancreatic NENs evaluated the effect of everolimus according to the degree of differentiation. The authors demonstrated that G3 pNECs and pNETs showed significantly shorter PFS compared to G1 and G2 NETs with the median PFS of 7.1 and 11.2 months, respectively, for G1 and G2 NETs vs. 1.4 and 3.6 months, respectively, G3 for NECs and NETs (HR 0.45; 95% CI: 0.26–0.78, *p* = 0.0005) [66].

A prospective phase II study also showed poor results with the use of everolimus in pNEC with a median PFS of 1.2 months, a median OS of 7.5 months and a DCR of 39.1% in patients treated with this drug as second-line therapy [75].

A more favorable response to everolimus is observed if the search is limited to well-differentiated G3 NETs only.

Panzuto et al. conducted a study on the use of everolimus in patients well-moderately differentiated G3 pNEC, enrolled before the formal introduction of G3 NET in the WHO classification. They identified a subgroup of patients with Ki-67 < 55% and with non-poorly differentiated morphology (NET G3 according to the most recent WHO classification), which has achieved encouraging results: a median PFS and OS of 6 and 28 months, respectively [76].

Promising results have recently been presented in the MAVERIC trial, a phase II randomized study, which evaluated the efficacy of everolimus as maintenance therapy in patients with GEP or lung NENs with Ki-67 between 20% and 55% at the end of the first-line chemotherapy. Patients who received everolimus showed significantly longer PFS compared to patients who underwent observation only (11.4 months vs. 1.8 months; *p* = 0.022) despite the OS was comparable (38.3 months and 38.2 months, respectively; *p* = 0.43). These data could contribute to the definition of a maintenance therapy in high-grade NENs, which are currently not available [77].

These data suggest that the efficacy of everolimus in high-grade forms is restricted mainly to G3 NETs, although with inferior results if compared to G1–G2 tumors, while, in aggressive poorly differentiated neuroendocrine forms, a targeted monotherapy has a very limited efficacy. Considering the poor prognosis due to the aggressive nature, further studies are needed to investigate the role of combined therapies with targeted agents and cytotoxic drugs in this setting.

As mentioned above, mTOR expression is higher in foregut tumors, suggesting a greater efficacy of mTOR inhibitors in these cases. An example is represented by the NEN of the thymus, a rare neoplasm burdened by a poor prognosis. M. Lang et al. described their experience with four patients with thymic NEN progressing after at least one line of treatment who were subsequently administered everolimus 10 mg/day. Disease stability was observed for a mean of 20.8 months with non-severe toxicity, suggesting a valid treatment proposal for thymic NENs in a second line of treatment, especially if the Ki-67 index is low [78].

Finally, the efficacy of immunotherapy has also been explored in the setting of neuroendocrine tumors, and several clinical trials are investigating the efficacy of various checkpoint inhibitors in patients with advanced/metastatic NETs.

Indeed, a non-randomized trial investigated the efficacy of pembrolizumab in patients with metastatic high-grade neuroendocrine neoplasms that had progressed after platinum-based chemotherapy; 3.4% of patients experienced an objective response, and the disease control rate was 24.1% [79]. Other studies have similarly demonstrated an antitumor activity of immunotherapy [80,81].

In murine models, the inhibition of mTORC1 and mTORC2 demonstrated a suppression of B lymphocyte activity, leading to a decrease in immunoglobulin synthesis and cellular proliferation. Moreover, a suppression of T-cell development into TH1, TH17 and Treg subtypes was seen, with an enhancement in the quality, functionality and effectiveness of memory T-cells, especially in reaction to viral stimuli [82]. Despite their immunosuppressive properties, mTOR inhibition engenders some advantageous immune regulatory actions that exhibit efficacy against diverse pathogens.

Moreover, everolimus, a mTORC1 inhibitor, has demonstrated antitumor efficacy in vitro and in murine models of T-cell lymphoma, indicating a possible function in enhancing the anti-tumor immune response [83].

Everolimus may affect the immunological response to vaccination, thereby diminishing the efficacy of immunizations administered during therapy. Consequently, the administration of live vaccinations is often discouraged during everolimus treatment [84].

This preclinical research suggests that mTOR inhibitors, like everolimus, may affect the immune response via intricate processes, encompassing both immunosuppressive and potentially immunostimulatory effects, contingent upon the context and individual cell types involved.

Nevertheless, at the moment, there are no studies of combination between everolimus and immunotherapy, which currently has no place in clinical practice since studies in the literature have several limitations, such as heterogeneous test populations.

## 8. Conclusions

Everolimus is an orally selective inhibitor of mTOR, which inhibits cell proliferation and angiogenesis. The RADIANT trials evaluated the effectiveness of this drug in NETs and led to the approval of everolimus in the treatment of G1–G2 NETs, with a good tolerability profile.

Many studies, mainly retrospective and with small and heterogeneous populations, have been published, aiming at identifying predictive biomarkers for everolimus efficacy. However, despite it having historically been an area of intense research, no predictive biomarkers have been validated.

In high-grade forms, the efficacy of everolimus is restricted mainly to G3 NETs, although with inferior results if compared to G1–G2 tumors. Instead, it has limited efficacy in the treatment of NECs.

Furthermore, the optimal treatment sequence in patients with advanced NETs has not been established. Everolimus is approved in pre-treated NETs, usually after SSA failure. Ongoing prospective clinical trials will hopefully define its ideal position in the therapeutic algorithm of NETs.

Several studies are underway to evaluate the use of everolimus in combination with other drugs such as anti-VEGF, anti-EGFR and anti-IGF1 monoclonal antibodies and immune checkpoint inhibitors.

## Figures and Tables

**Table 1:** Summary of radiant trials.

	RADIANT 1	RADIANT 2	RADIANT 3	RADIANT 4
Phase	II	III	III	III
Number of patients	160	429	410	302
Setting	Metastatic, progressive, pancreatic NET, G1–G2	Advanced, progressive, functional NETs with GI, pulmonary or unknown origin, G1–G2	Advanced, progressive, pancreatic NET, G1–G2	Advanced, progressive, non functional lung or GI NET, G1–G2
Treatment	Everolimus 10 mg (1st group) vs. everolimus 10 mg + octreotide (2nd group)	Everolimus 10 mg + octreotide LAR 30 mg (1st group) vs. placebo + octreotide LAR 30 mg (2nd group)	Everolimus 10 mg (1st group) vs. placebo (2nd group)	Everolimus 10 mg (1st group) vs. placebo (2nd group)
Primary endpoint	ORR in 1st group	PFS	PFS	PFS
PFS	9.7 months in 1st group, 16.7 months in 2nd group	16.4 months in 1st group vs. 11.3 in 2nd group	11 months in 1st group vs. 4.6 months in 2nd group	11 months in 1st group vs. 3.9 months in 2nd group
OS	OS not reached in 1st group vs. 24.9 months in 2nd group	No difference between 1st and 2nd groups	No difference between 1st and 2nd groups	No difference between 1st and 2nd groups

## Data Availability

The data can be shared on request.
